# Food availability and affordability in a Mediterranean urban context: associations by store type and area-level socio-economic status

**DOI:** 10.1017/S1368980022002348

**Published:** 2022-10-24

**Authors:** Carlos Fernández-Escobar, Julia Díez, Alba Martínez-García, Usama Bilal, Martin O’Flaherty, Manuel Franco

**Affiliations:** 1 National School of Public Health, Institute of Health Carlos III, Madrid, Spain; 2 Public Health and Epidemiology Research Group, Faculty of Medicine and Health Sciences, University of Alcalá, Alcalá de Henares, Spain; 3 Department of Community Nursing, Preventive Medicine and Public Health and History of Science, University of Alicante, 03690 Alicante, Spain; 4 Urban Health Collaborative and Department of Epidemiology and Biostatistics, Dornsife School of Public Health, Drexel University, Philadelphia, PA, USA; 5 Department of Public Health, Policy and Systems, University of Liverpool, Liverpool, UK; 6 Department of Epidemiology, Johns Hopkins Bloomberg School of Public Health, Baltimore, MD, USA

**Keywords:** Food environment, NEMS-S-MED, Food availability, Food prices

## Abstract

**Objective:**

Although food environments have been highlighted as potentially effective targets to improve population diets, evidence on Mediterranean food environments is lacking. We examined differences in food availability and affordability in Madrid (Spain) by store type and area-level socio-economic status (SES).

**Design:**

Cross-sectional study. Trained researchers conducted food store audits using the validated Nutrition Environment Measures Survey in Stores for Mediterranean contexts (NEMS-S-MED) tool to measure the availability and price of twelve food groups (specific foods = 35). We computed NEMS-S-MED scores and summarised price data with a Relative Price Index (RPI, comparing prices across stores) and an Affordability Index (normalising prices by area-level income). We compared the availability and affordability of ‘healthier–less healthy’ food pairs, scores between food store types (supermarkets, specialised, convenience stores and others) and area-level SES using ANOVA and multi-level regression models.

**Setting:**

City of Madrid. 2016 and 2019 to cover a representative sample.

**Participants:**

Food stores within a socio-economically diverse sample of sixty-three census tracts (*n* 151).

**Results:**

Supermarkets had higher food availability (37·5/49 NEMS-S-MED points), compared to convenience stores (13·5/49) and specialised stores (8/49). Supermarkets offered lower prices (RPI: 0·83) than specialised stores (RPI: 0·97) and convenience stores (RPI: 2·06). Both ‘healthy’ and ‘less healthy’ items were more available in supermarkets. We found no differences in food availability or price by area-level SES, but affordability was higher in higher-income areas.

**Conclusions:**

Supermarkets offered higher food availability and affordability for healthy and less healthy food items. Promoting healthy food availability through supermarkets and specialised stores and/or limiting access to convenience stores are promising policy options to achieve a healthier food environment.

Unhealthy diets are the leading risk factor of non-communicable diseases morbidity and mortality^(1)^ and contribute to socio-economic inequities^(2)^. Food environments are defined as ‘the collective physical, economic, political and socio-cultural circumstances surrounding population’s food/beverage options and nutritional status’^(3)^. Specifically, Glanz et al. conceptualised the retail food environment as encompassing both the ‘community food environment’ (physical access to food outlets) and the ‘consumer food environment’ (availability of healthy food, price, promotion and placement within outlets)^(4)^. Given their potential role in shaping food access within people’s living and working environments, food environments have received growing scientific and political attention over the last decades^(5–7)^.

Previous research has assessed the evidence between the consumer food environment and dietary/health outcomes; however, the associations varied^(8–10)^. This lack of consistency may be partly due to three issues. First, affordability has been often unaccounted for^(6,11)^. Second, more evidence is needed to understand the interaction between the consumer food environment and area-level socio-economic status (SES). Although socio-economically disadvantaged urban areas have shown greater accessibility to unhealthy foods, this relationship varies when considering affordability of healthy foods^(12–14)^. Third, consumer food environments are context-dependent and vary between countries, cities or neighbourhoods^(15)^. Yet, current evidence is still focussed on Anglo-Saxon settings like the USA, Australia or Canada^(6)^. The lack of evidence in Southern Europe^(16,17)^ is a key shortcoming for the identification of the effects of exposure to Mediterranean food environment^(18)^.

To fill these gaps, this study aimed to examine differences in availability and affordability of different food products in the city of Madrid (Spain) by store type and area-level SES.

## Methods

### Study design and sample

This study was part of the Heart Healthy Hoods project, which analysed the relationship between the socio-physical urban environment and cardiovascular health in Madrid, Spain^(19)^. Madrid is organised into twenty-one administrative districts, which are divided into 129 neighbourhoods, and into 2443 census tracts—the smallest administrative areas in Madrid, with a median population of 1500 residents. We used a multistage design to sample diverse areas (see online supplementary material, Supplemental Fig. S1). We sampled three neighbourhoods (high-, middle- and low-SES) per district and selected median census tracts in each neighbourhood in terms of socio-economic characteristics (*n* 63). Sampling strategy has been described in more detail elsewhere^(20,21)^.

### Data collection

We conducted store audits using the ‘Nutrition Environment Measures Survey in Stores for Mediterranean contexts’ (NEMS-S-MED) tool^(21)^. The original NEMS-S^(22)^, designed for the US context, is one of the most widely used tools for conducting food store audits^(23)^. The adapted NEMS-S-MED tool evaluates availability and price within twelve food groups: fresh fruits, vegetables, nuts, non-alcoholic beverages, bread, cereals and bakery, milk and dairy products, eggs, oil and butter, rice, legumes, meat and fish. We also recorded the availability of alcoholic beverages. Data collection occurred in two waves in June–July 2016 and November–December 2019 (see online supplementary material, Supplemental Fig. S2), on weekdays and during business hours. Trained observers audited all food stores located within each census tract, assessing and scoring each measure following a standard protocol. We integrated the NEMS-S-MED audit tool into the Open Data Kit app for Android smartphones to facilitate data collection. More details are available elsewhere^(21)^.

### Measures

We measured the availability of food by the presence of selected food items within twelve food groups (i.e. apples in the ‘fresh fruit’ group) (Table A1). NEMS-S-MED score ranges from 0 to 49, with higher scores representing higher availability and variety and lower prices of healthier food options^(21)^. We recorded price (either per grams or per item if sold only by the piece) of selected items, to be compared between food stores. All prices reflect non-sale price. NEMS-S-MED tool is available elsewhere^(21)^.

We categorised food stores into supermarkets (including discounters), convenience stores (including corner stores and gas stations) and traditional/specialised stores (fruits and vegetables stores, butcheries, fishmongers and bakeries) based on previous research^(21,24)^. We excluded food markets and food galleries. Food markets in Spain are a collection of tens of stalls mostly dedicated to retailing a single category of foods (e.g. fruits/vegetables, fish, meat, bakery products, etc.). Standard tools for healthy food availability measures can fail to capture the effect of these retailers^(19)^.

To measure area-level SES, we used a validated composite index at census tract level^(25)^. This SES index is constructed from seven indicators: (1) low education, (2) high education, (3) part-time employment, (4) temporary employment, (5) manual occupational class, (6) average housing prices and (7) unemployment rate. Further details are available elsewhere^(25)^. We operationalised this measure into quintiles (Q1 = most socio-economically disadvantaged and Q5 = most socio-economically advantaged) using data from 2017. We also obtained the census tract mean income per capita from the National Institute of Statistics^(26)^.

### Statistical analyses

We calculated descriptive statistics of availability and price by food store type and area-level SES (quintiles). We compared availability and price of ‘healthier – less healthy’ food pairs and tested for differences using two-sample test of proportions and Wilcoxon matched-pairs signed-rank test, respectively.

We summarised price data calculating a Relative Price Index (RPI) for each store:

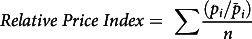




Where 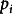 is the price of a food item, 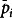 is the mean price of that item across all stores in our sample and 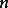 is the total number of food items with recorded price in the store. This allowed us to compare between food stores that sold a different number of distinct products (e.g. a fruit and vegetables store *v*. a supermarket).

To account for different purchasing power of residents, we also computed an Affordability Index for each food store, dividing the relative mean income per capita of its census tract by the RPI of the retailer:

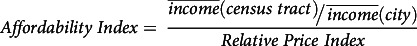




Income (city) refers to the mean income per capita in Madrid and was sourced directly from the National Statistics Institute^(26)^. A higher Affordability Index means that the food store is more affordable, considering the mean income of their census tract.

We computed NEMS-S-MED score, RPI and Affordability Index for each store. We compared these metrics by store type and quintile of area-level SES using ANOVA. In addition, we fitted a multilevel regression model of stores nested in census tracts, including a fixed effect for store type, area-level SES and year of data collection. Data analysis was conducted with Stata 15 (StataCorp, 2017).

## Results

### Descriptive data

We audited 151 food retailers. Almost half of them (45·0 %, *n* 68) were convenience stores, followed by specialised stores (30·5 %, *n* 46), supermarkets (18·5 %, *n* 28) and others (6·0 %, *n* 9), the latter including coffee shops and herbalists selling food products.

Table 1 displays food availability of NEMS-S-MED food items. Most frequent available items were eggs, alcoholic drinks, cola drinks, not-100 % juice, legumes, cold meat and milk, each of them with an availability greater than 70 %. Least common items were fresh fish (13·3 %), whole rice (17·3 %), processed and unprocessed frozen fish (18·0 % and 20·0 %, respectively), chicken and beef (19·3 % each) and frozen vegetables (21·3 %). Mean availability across all food items was 89·7 % in supermarkets, 60·1 % in convenience stores, 22·5 % in specialised stores and 32·1 % in other stores. The biggest difference in availability between supermarkets and convenience stores was for fresh meat, fish, fresh fruit and whole rice. Supermarkets had similar availability of fruit and vegetables, meat and bread than fruit and vegetables stores, butchers and bakeries, respectively (Table A2). However, fresh and unprocessed frozen fish had lower availability in supermarkets (57·1 % and 78·6 %, respectively) *v*. fishmongers (100 %). ‘Less healthy’ alternatives (e.g. salty nuts, not-100 % juice, cola drinks, sugary cereals, cold meat and confectionery) were more available in supermarkets than in specialised stores.

**Table 1:** Availability of food items by type of food store

Food item	Type of retailer					
	All (*n* 151)	Supermarket (*n* 28)	Convenience store (*n* 68)	Specialised store (*n* 46)	Other (*n* 9)	*P*-value*
Fresh fruit	56·3	100	47·1	47·8	33·3	<0·01
Fresh vegetables	60·9	100	57·4	47·8	33·3	<0·01
Frozen vegetables	21·2	75·0	10·3	4·3	22·2	<0·01
Unprocessed nuts	55·6	89·3	61·8	26·1	55·6	<0·01
Salty nuts	69·5	96·4	89·7	28·3	44·4	<0·01
Juice 100 %	37·1	78·6	38·2	8·7	44·4	<0·01
Not-100 % juice	73·5	100	94·1	32·6	44·4	<0·01
Light cola drink	75·5	96·4	100	34·8	33·3	<0·01
Regular cola drink	74·8	96·4	98·5	34·8	33·3	<0·01
Whole bread	54·3	89·3	57·4	34·8	22·2	<0·01
Low sugar cereals	31·8	78·6	30·9	4·3	33·3	<0·01
Regular cereals	43·0	92·9	54·4	2·2	11·1	<0·01
Skimmed milk	71·5	96·4	97·1	26·1	33·3	<0·01
Semi-skimmed milk	71·5	96·4	97·1	28·3	22·2	<0·01
Whole milk	72·2	96·4	97·1	28·3	33·3	<0·01
Skimmed yogurt	37·7	89·3	38·2	6·5	33·3	<0·01
Cream cheese	43·7	92·9	47·1	8·7	44·4	<0·01
Semi-hard cheese	58·3	92·9	75·0	13·0	55·6	<0·01
Eggs	79·5	96·4	92·6	56·5	44·4	<0·01
Olive oil	53·6	85·7	67·6	19·6	22·2	<0·01
Sunflower oil	65·6	100	86·8	19·6	33·3	<0·01
Salt-free butter	35·8	89·3	38·2	4·3	11·1	<0·01
Regular butter	50·3	92·9	63·2	13·0	11·1	<0·01
Whole rice	17·2	60·7	8·8	2·2	22·2	<0·01
White rice	68·2	96·4	91·2	21·7	44·4	<0·01
Legumes	73·5	100	86·8	41·3	55·6	<0·01
Potatoes	58·3	100	52·9	43·5	44·4	<0·01
Chicken	19·9	75·0	1·5	10·9	33·3	<0·01
Beef	19·9	75·0	2·9	10·9	22·2	<0·01
Cold meat	72·8	96·4	92·6	32·6	55·6	<0·01
Fresh fish	13·2	57·1	0·0	6·5	11·1	<0·01
Unprocessed frozen fish	20·5	78·6	7·4	6·5	11·1	<0·01
Processed frozen fish	18·5	71·4	8·8	2·2	11·1	<0·01
Canned tuna	68·2	96·4	94·1	23·9	11·1	<0·01
Confectionery	66·9	100	76·5	39·1	33·3	<0·01

* ANOVA.

Table A3 shows a comparison of pairs of ‘healthy-less healthy’ alternatives. Most healthier food items were less available than their less healthy counterparts, e.g. juice 100 % than not-100 % juice, low sugar cereals than regular ones, virgin olive oil than refined sunflower oil or whole rice than white rice. There were no differences in availability between light and regular cola drinks, skimmed milk and whole milk, chicken and beef or unprocessed and processed frozen fish. Some healthier items were more expensive than their less healthy pairs (olive oil, whole rice and 100 % juice), one item (chicken) was cheaper and several pairs had a similar price (cola drinks, cereals and milk).

### NEMS-S-MED Score

**Fig. 1:**
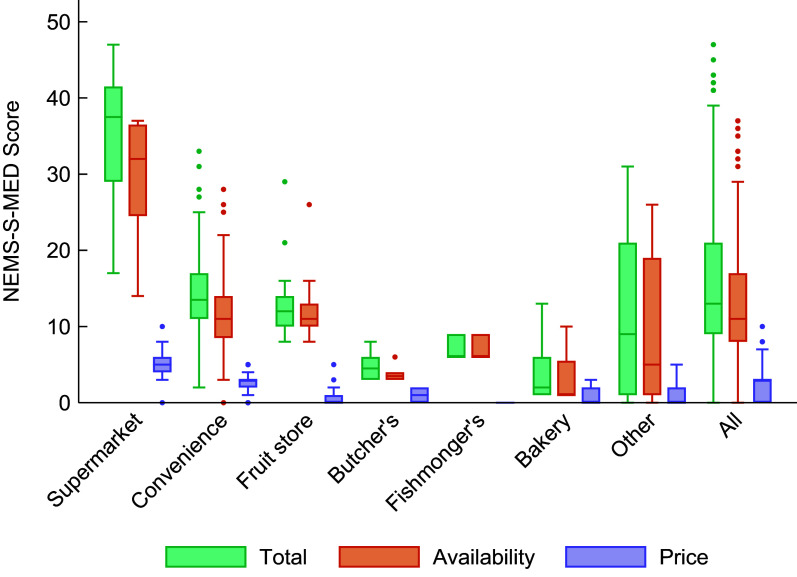
NEMS-S-MED scores by store type. NEMS-S-MED, Nutrition Environment Measures Survey in Stores for Mediterranean contexts

We found a large variability in NEMS-S-MED scores by type of retailer, with an overall median of thirteen out of a total of forty-nine points (interquartile range, IQR: 12) (Fig. 1 and Table A4). Supermarkets had the highest total score (median: 37·5, IQR:12·5), followed by small/convenience stores (median: 13·5, IQR: 6). As a group, specialised stores scored a median of eight out of forty-nine points. Of those, fruit stores had the highest availability (median: 11, IQR: 3).

### Price

The RPI ranged from 0·52 (price of food items was 48 % lower than average) to 4·22 (price of food items was 322 % higher than average). As seen in Table A5, fishmongers had the lowest RPI, although it accounted only for the price of hake, and the difference with supermarkets was NS. Apart from fishmongers, supermarkets had the lowest overall RPI (median 0·83, IQR 0·33), followed by other specialised stores (median ranging from 0·96 to 0·98). Convenience stores were more expensive than supermarkets (median 1·02, IQR 0·26), and retailers in the ‘other’ category were the most expensive of all (median 2·06, IQR 1·13). Supermarkets had significantly lower prices in fresh fruits and vegetables, cola drinks, regular cereals, milk, sunflower oil, white rice and beef compared to convenience stores (Table A6). Prices of fruit and vegetables was similar between supermarkets and fruit and vegetables stores (*P* = 0·54) (Table A7).

### Area-level socio-economic differences

Table 2 shows number of food retailers, median values of NEMS-S-MED scores and mean values of RPI, Affordability Index and SES Index across quintiles of SES. Median number of food stores was similar between SES quintiles (*P* = 0·84). Median total NEM-S-MED scores went from 12 to 18, without significant differences (*P* = 0·39). The same was true for RPI (mean 0·95 to 1·24, *P* = 0·18). Affordability Index increased monotonically with SES, indicating higher affordability in higher SES areas (*P* < 0·01).

**Table 2:** Differences in number of food retailer, NEMS-S-MED scores and selected indexes across quintiles of SES

	Census tract SES Quintile										
	Low (*n* 11)		Low-medium (*n* 8)		Medium (*n* 10)		Medium-high (*n* 12)		High (*n* 11)		*P*-value*
	Median	Interquartile range	Median	Interquartile range	Median	Interquartile range	Median	Interquartile range	Median	Interquartile range	
Number of food stores†	2	3	3·5	3·5	3	3	2	2	2	3	0·84
Supermarkets	0	0	1	2	1	1	0	1	0	1	0·12
Convenience stores	2	3	1	1	1	1	1	0·5	1	1	0·44
Specialised stores	0	1	1	1	0	1	0	0·5	0	0	0·34
Others	0	0	0	0	0	0	0	0	0	0	0·63
NEMS-S-MED Score†											
Total (0–49)	12	3·5	18	14·25	13·5	6·5	17·5	14·75	18	11·5	0·39
Availability (0–37)	9	2·5	15·5	11	10·5	4	14	12·5	14	9·5	0·30
Price (0–12)	3	0	2·5	2·25	2·5	3	3	1	3	3·5	0·84
	Mean	sd	Mean	sd	Mean	sd	Mean	sd	Mean	sd	
SES Index‡	−1·19	0·19	−0·62	0·17	−0·03	0·24	0·55	0·13	1·06	0·22	<0·01
Price Index‡	0·95	0·15	1·06	0·23	1·02	0·29	0·94	0·15	1·24	0·56	0·18
Affordability Index‡	0·66	0·19	0·83	0·27	0·96	0·16	1·28	0·26	1·29	0·37	<0·01

* ANOVA.NEMS-S-MED, Nutrition Environment Measures Survey in Stores for Mediterranean contexts; SES, socio-economic status.

† Median (Interquartile range).

‡ Mean (sd).

Table 3 shows results from a multi-level linear regression model. Across all dependent variables (NEM-S-MED scores, RPI and Affordability Index), most of the variability was at store level as compared to census tract level (Intraclass Correlation = 23 %, 17 %, 17 %, 13 % and 33 % for total NEMS-S-MED score, availability score, price score, RPI and Affordability Index, respectively). All store types had lower NEMS-S-MED total, availability and price scores than supermarkets (*P* < 0·01). Supermarkets showed the lowest prices, although the difference was only significant when comparing supermarkets to bakeries and ‘other’ stores. All store types but fishmongers were less affordable than supermarkets. NEM-S-MED scores were not different between SES quintiles. There were RPI differences across SES quintiles for the quintile with the highest index, which showed a RPI 0·38 points higher than the low-SES quintile (95 % CI: 0·13, 0·64, *P* < 0·05). No other differences were observed by SES. Affordability Index was associated with census tract-level SES, showing higher affordability in high-income areas (*P* < 0·01).

**Table 3:** Multilevel linear regression model for NEM-S-MED scores, relative price index and affordability index

Parameter	NEMS-S-MED total		NEMS-S-MED availability		NEMS-S-MED price		Price Index		Affordability Index	
Fixed effects	Estimate	95 % CI	Estimate	95 % CI	Estimate	95 % CI	Estimate	95 % CI	Estimate	95 % CI
Type of food retailer (Base: Supermarket)										
Convenience store	**−20·24** **	–22·89, –17·59	**−17·66** **	–19·99, –15·33	**−2·52** **	–3·15,–1·89	0·17	–0·01, 0·35	**−0·27** **	–0·39,–0·15
Fruit and vegetables	**−22·08** **	–25·39, –18·77	**−17·64** **	–20·56, –14·72	**−4·44** **	–5·22,–3·66	0·09	–0·13, 0·31	**−0·16** *	–0·32,–0·00
Butcher’s	**−30·96** **	–36·11, –25·81	**−26·46** **	–31·03, –21·89	**−4·51** **	–5·73,–3·29	0·24	–0·09, 0·57	**−0·26** *	–0·50,–0·02
Fishmonger’s	**−27·17** **	–34·05, –20·29	**−22·22** **	–28·32, –16·12	**−5·14** **	–6·77,–3·51	0·01	–0·44, 0·46	−0·07	–0·38, 0·24
Bakery	**−29·25** **	–32·84, –25·66	**−25·26** **	–28·44, –22·08	**−4·15** **	–4·99,–3·31	**0·83** **	0·54, 1·12	**−0·42** **	–0·64,–0·20
Other	**−23·52** **	–28·01, –19·03	**−19·47** **	–23·43, –15·51	**−4·08** **	–5·14,–3·02	**0·41** *	0·04, 0·78	**−0·43** **	–0·68,–0·18
SES Index quintile (Base: Low)										
Low-medium	1·70	–2·57, 5·97	1·82	–1·73, 5·37	−0·06	–1·00, 0·88	0·16	–0·11, 0·43	0·10	–0·12, 0·32
Medium	4·17	0·07, 8·27	**3·71** *	0·30, 7·12	0·52	–0·38, 1·42	0·12	–0·13, 0·37	**0·24** *	0·02, 0·46
Medium-high	3·24	–1·13, 7·61	3·01	–0·62, 6·64	0·07	–0·89, 1·03	0·18	–0·09, 0·45	**0·51** **	0·29, 0·73
High	3·68	–0·34, 7·70	3·37	0·02, 6·72	0·18	–0·72, 1·08	**0·38** **	0·13, 0·63	**0·61** **	0·41, 0·81
Year (Base: 2016)	−1·80	–4·74, 1·14	−1·25	–3·68, 1·18	−0·50	–1·15, 0·15	−0·11	–0·31, 0·09	0·06	–0·10, 0·22
Random effects										
Census tract-level variance	8·93		5·03		0·36		0·02		0·03	
Store-level variance	30·12		24·02		1·70		0·13		0·06	
Intraclass correlation	0·23		0·17		0·17		0·13		0·33	

NEMS-S-MED, Nutrition Environment Measures Survey in Stores for Mediterranean contexts; SES, socio-economic status. Boldface indicates statistical significance

* *P* < 0·05,

** *P* < 0·01.

Intraclass correlation = census tract-level variance/(census tract-level variance + store-level variance).

## Discussion

This study evaluated the consumer food environment in Madrid, Spain, an example of a Southern European/Mediterranean urban context. We report three key findings. First, we found that food availability and affordability were greater in supermarkets and specialised stores than in convenience stores. Second, we found no socio-economic inequities in the number of food stores per census tract or healthy food availability. Third, we found that in higher-income areas food prices were above average, although affordability was also higher than in lower-income areas.

Previous research in other countries has found a higher availability and affordability of healthy food in supermarkets and larger stores than in smaller stores^(16,27–29)^. However, this is also true for ultra-processed foods and other unhealthy foods, as we have also seen in our data, making supermarkets a ‘double-edged sword’^(30)^. As other studies have shown, product placement strategies in supermarkets promote higher sales and consumption of both healthy and unhealthy foods^(31)^. Supermarkets are the main source of foods and beverages purchased for home consumption in Spain, accounting for 61·4 % of all Spain retail grocery sales in 2019^(32)^. However, small specialised stores still contribute to approximately 30 % of all fresh food sales^(32)^.

Due to the political and economic implications that supermarkets have on the food systems and the retail food environment^(33,34)^, and their prominence in Spanish local food environments in terms of food availability, food prices and high sales volume, supermarkets are a desirable target for health-promoting policies. In this regard, supermarket and food environment, actions supporting healthy and sustainable diets would be to ensure the affordability and availability of a variety of healthy foods, along with the reduction or withdrawal on unhealthy products such as confectionery, snacks and sugary drinks, in addition to the development of interventions that encourage healthy food choices by consumers^(33–35)^.

On the other hand, traditional specialised stores offered high fresh food availability at comparable prices to supermarkets, with the added benefit of lower availability of unhealthy products (i.e. alcoholic drinks or confectionery). Although replacement of traditional shops with supermarkets may have deleterious effects overall by moving diets towards a more Western pattern, high in ultra-processed foods^(30)^, some authors advocate for increasing the number of supermarkets, specially in deprived or underserved neighbourhoods^(14)^. We found that supermarkets were as prevalent as specialised stores in census tracts (usually 0–1 supermarkets and 0–1 specialised stores per census tract), but several different specialised stores are needed to offer a viable alternative for supermarkets in terms of fresh food availability (e.g. at least a fruit store, a butchers and a fishmongers). Increasing the number and variety of traditional stores may be a promising strategy to promote fresh product purchases^(14,36)^. Alternatively, public food markets, which include multiple stalls of a diverse set of specialised stores, may also increase healthy food availability. City governments can use their licencing powers to ensure the presence of a variety of traditional stores in all neighbourhoods. These local actions should be supported by the local and national governments through the development of policies and interventions promoting healthy retail food environments, as well as encouraging fair, local and proximity trade^(37)^.

Convenience stores in our sample offered less varied and more expensive food items. From a public health perspective, their widespread presence might be detrimental in local food environments, as they usually lack varied, affordable fresh products and most food offered are high-calorie, easy-to-preserve items. Proximity to convenience stores in the USA has been associated with higher prevalence of obesity^(38)^. Local interventions that limit access to convenience stores (e.g. via retailer licensing) might be considered^(14)^. In-store interventions which combine price, engaging information and easier access to and availability of healthy foods are also promising strategies^(39)^.

In contrast to previous literature set in countries like the USA^(40)^ or Brazil^(41)^, we did not find evidence of ‘food deserts’, or areas in deprived neighbourhoods with low to no access to healthy foods. This is probably due to stark differences in urban planning and food culture across European and American cities. For example, in a previous study, we found that more than three-quarters of residents in a Madrid neighbourhood lived nearer than 200 m from a food store with healthy food, in contrast to more than 95 % of Baltimore’s resident living farther than 400 m from these stores^(15)^. This discrepancy also highlights the need to tailor urban food policies to their specific environment and suggests that some recommended interventions, such as actions to improve availability in food deserts^(42)^, may be of no use in Mediterranean contexts. However, this aspect should be further studied in other Southern European and Mediterranean context.

We found a homogeneous consumer food environment across all SES quintiles, except for the quintile with the highest index, and most of the variability in availability and affordability scores was at store level. These results are in accordance with recent research conducted in Malta^(27)^, Australia^(29)^ and the United Kingdom^(28)^. However, similar prices across the geography can regardless mean a higher financial burden for low-income families, as our Affordability Index showed. This suggests that affordability, and not availability, may be the most important driver of dietary socio-economic inequities in Mediterranean contexts. Previous literature has shown that healthy foods are considerably more expensive in terms of price per weight or per calorie^(11)^. In this regard, policy interventions in food prices are recommended as one of the most potentially effective public health policies promoting healthy diets^(43)^. Either an overall decrease in healthy food prices or a targeted price discount for low-income populations living in ‘affordability deserts’ may help reduce health inequities. It is important to bare in mind the current income inequality trends in our cities and countries, therefore income and wealth redistribution policies are also relevant pieces for reducing dietary inequities.

We also found that healthier food alternatives, such as whole rice instead of white rice, or virgin olive oil instead of refined sunflower oil, are usually less available and costlier. This may represent a hurdle for low-income individuals and families to switch from less to more healthy diets. Public incentives to reduce price of healthier alternatives, e.g. subsidies for olive oil or whole rice producers, coupled with disincentives towards unhealthy elements (e.g. a sugar tax) are promising policy options to improve consumer food environments.

### Study limitations and strengths

This is, to our knowledge, the first study evaluating the consumer food environment in a large city in a Mediterranean context. We sampled a wide variety of socio-economic backgrounds using reproducible and scalable methods. We used a validated novel audit tool which allowed us to describe and compare both food availability and affordability of culturally relevant food items.

Several limitations of our study should be considered. First, some differences in food availability and affordability may exist that we failed to detect due to a possibly small sample. We used a convenience, non-random sample, so extrapolation of results should be cautious, although we selected our sample to guarantee socio-economic diversity. Second, data collection took place in two discrete time periods, so we cannot evaluate possible seasonal effects or trends in the consumer food environment, although we did not find significant differences between the two data collection points. Third, we focussed on store food availability but we lacked direct data on consumer purchases or intake. Furthermore, important aspects of the consumer food environment are missing in NEMS-S-MED, such as presence of marketing campaigns, relative shelf space of different products, food quality or availability of ultra-processed foods. We also excluded food markets and galleries from our sample. However, the audit tool offers a reasonable compromise between comprehensiveness and practicality, has been previously validated in our context and can be easily replicated in other Mediterranean cities.

Our results might be cautiously extrapolated to other high-density Mediterranean cities. Madrid has a higher income, more educated population than the rest of Spain, and is more ethnically diverse with larger socio-economic inequalities^(44)^. Smaller, more homogenous cities may exhibit even more consistent consumer food environments. Rural areas or cities with prominent urban sprawls might present greater differences in food availability and access by SES, as is the case with large American cities.

## Conclusions

In our study of the consumer food environment of the city of Madrid, we found that supermarkets had greater food availability and overall lower prices than specialised and convenience stores, for both healthy and less healthy foods. We found no differences in food availability or price by area-level SES, and a higher relative affordability for high-income areas. Further studies should explore the generalisability of these results to other European/Mediterranean cities. Promoting healthy food availability through supermarkets and specialised stores and/or limiting access to convenience stores are promising policy options to achieve a healthier consumer food environment.

## Supplementary material

For supplementary material accompanying this paper visit https://doi.org/10.1017/S1368980022002348. click here to view supplementary material
