# Supplementary material for: Food availability and affordability in a Mediterranean urban context: associations by store type and area-level socio-economic status
Source: Public Health Nutr. 2022 Oct 24;26(2):446–54. doi: 10.1017/S1368980022002348 (PMC13076083; doi:10.1017/S1368980022002348)

# Supplementary material

**Table A1. Food categories and items included in the NEMS-S-MED audit tool**

| **Food item** | **Availability** | **Price** ^1^ | |
| --- | --- | --- | --- |
|  |  | **Absolute** | **Comparative** |
| 1. Fresh fruits |  |  |  |
| - Fresh fruit varieties | X |  |  |
| - Apples | X | X |  |
| 2. Vegetables (fresh/frozen) |  |  |  |
| - Fresh vegetable varieties | X |  |  |
| - Tomatoes | X | X |  |
| - Frozen spinach | X | X |  |
| - Potatoes | X |  |  |
| 3. Nuts |  |  |  |
| - Raw vs. processed | X |  |  |
| 4. Non-alcoholic beverages |  |  |  |
| - Soda: Diet Coke vs. regular Coke | X |  | X |
| - Fruit juice: 100% juice vs. juice drinks | X |  | X |
| 5. Bread, cereals, and baked goods |  |  |  |
| - 100% whole grain bread | X |  |  |
| - Baked goods | X |  |  |
| - Plain cereals vs. >5 g of sugar per 100 g | X |  | X |
| 6. Milk and dairy products |  |  |  |
| - Skim/low-fat vs. whole milk | X |  | X |
| - White cheese vs. aged cheese | X |  |  |
| - Low-fat yoghurts | X |  |  |
| 7. Eggs | X |  |  |
| 8. Oil and butter | X |  |  |
| - Extra virgin olive oil | X | X |  |
| - Sunflower oil | X | X |  |
| - Butter vs. non-added salt butter | X |  |  |
| 9. Rice |  |  |  |
| - Whole rice vs. white rice | X |  | X |
| 10. Legumes | X |  |  |
| 11. Meat and meat products |  |  |  |
| - Red meat (beef) vs. poultry (chicken) | X |  | X |
| - Processed meat | X |  |  |
| 12. Fish |  |  |  |
| - Fresh fish varieties | X | X |  |
| - Fresh hake | X | X |  |
| - Unprocessed vs. processed seafood | X |  |  |
| - Canned tuna | X |  |  |

^1^ Following Glanz et al. [**22**], absolute price applies when the item is compared across store type and neighborhood characteristics, while comparative price applies when there is price information for a healthier option and the “regular” comparison (e.g., diet vs. regular soda).

**Table A2. Availability of individual food items by store type, detail.**

| **Food item** | **Availability (% by type of retailer)** | | | | | | | | **p-value*** |
| --- | --- | --- | --- | --- | --- | --- | --- | --- | --- |
|  | **Total** | **Supermarket (n=28)** | **Convenience (n=68)** | **F&Vstore (n=21)** | **Butcher's' (n=6)** | **Fishmonger's' (n=3)** | **Bakery (n=16)** | **Other (n=9)** |  |
| Fresh fruit | 56.3 | 100 | 47.1 | 100 | - | - | 6.3 | 33.3 | <0.01 |
| Fresh vegetables | 60.9 | 100 | 57.4 | 100 | - | - | 6.3 | 33.3 | <0.01 |
| Frozen vegetables | 21.2 | 75.0 | 10.3 | 4.8 | - | 33.3 | - | 22.2 | <0.01 |
| Unprocessed nuts | 55.6 | 89.3 | 61.8 | 47.6 | - | - | 12.5 | 55.6 | <0.01 |
| Salty nuts | 69.5 | 96.4 | 89.7 | 33.3 | - | - | 37.5 | 44.4 | <0.01 |
| Juice 100% | 37.1 | 78.6 | 38.2 | 19.0 | - | - | - | 44.4 | <0.01 |
| Not-100% juice | 73.5 | 100 | 94.1 | 33.3 | - | - | 50.0 | 44.4 | <0.01 |
| Light cola drink | 75.5 | 96.4 | 100 | 28.6 | - | - | 62.5 | 33.3 | <0.01 |
| Regular cola drink | 74.8 | 96.4 | 98.5 | 28.6 | - | - | 62.5 | 33.3 | <0.01 |
| Whole bread | 54.3 | 89.3 | 57.4 | 9.5 | - | - | 87.5 | 22.2 | <0.01 |
| Low sugar cereals | 31.8 | 78.6 | 30.9 | 4.8 | - | - | 6.3 | 33.3 | <0.01 |
| Regular cereals | 43.0 | 92.9 | 54.4 | 4.8 | - | - | - | 11.1 | <0.01 |
| Skimmed milk | 71.5 | 96.4 | 97.1 | 33.3 | - | - | 31.3 | 33.3 | <0.01 |
| Semi-skimmed milk | 71.5 | 96.4 | 97.1 | 33.3 | - | - | 37.5 | 22.2 | <0.01 |
| Whole milk | 72.2 | 96.4 | 97.1 | 33.3 | - | - | 37.5 | 33.3 | <0.01 |
| Skimmed yogurt | 37.7 | 89.3 | 38.2 | 14.3 | - | - | - | 33.3 | <0.01 |
| Cream cheese | 43.7 | 92.9 | 47.1 | 14.3 | 16.7 | - | - | 44.4 | <0.01 |
| Semi-hard cheese | 58.3 | 92.9 | 75.0 | 14.3 | 33.3 | - | 6.3 | 55.6 | <0.01 |
| Eggs | 79.5 | 96.4 | 92.6 | 76.2 | 66.7 | - | 37.5 | 44.4 | <0.01 |
| Olive oil | 53.6 | 85.7 | 67.6 | 19.0 | - | - | 31.3 | 22.2 | <0.01 |
| Sunflower oil | 65.6 | 100 | 86.8 | 28.6 | - | - | 18.8 | 33.3 | <0.01 |
| Salt-free butter | 35.8 | 89.3 | 38.2 | 9.5 | - | - | - | 11.1 | <0.01 |
| Regular butter | 50.3 | 92.9 | 63.2 | 19.0 | - | - | 12.5 | 11.1 | <0.01 |
| Whole rice | 17.2 | 60.7 | 8.8 | 4.8 | - | - | - | 22.2 | <0.01 |
| White rice | 68.2 | 96.4 | 91.2 | 28.6 | - | - | 25.0 | 44.4 | <0.01 |
| Legumes | 73.5 | 100 | 86.8 | 61.9 | 33.3 | - | 25.0 | 55.6 | <0.01 |
| Potatoes | 58.3 | 100 | 52.9 | 95.2 | - | - | - | 44.4 | <0.01 |
| Chicken | 19.9 | 75.0 | 1.5 | - | 83.3 | - | - | 33.3 | <0.01 |
| Beef | 19.9 | 75.0 | 2.9 | - | 83.3 | - | - | 22.2 | <0.01 |
| Cold meat | 72.8 | 96.4 | 92.6 | 33.3 | 83.3 | - | 18.8 | 55.6 | <0.01 |
| Fresh fish | 13.2 | 57.1 | - | - | - | 100 | - | 11.1 | <0.01 |
| Unprocessed frozen fish | 20.5 | 78.6 | 7.4 | - | - | 100 | - | 11.1 | <0.01 |
| Processed frozen fish | 18.5 | 71.4 | 8.8 | - | - | 33.3 | - | 11.1 | <0.01 |
| Canned tuna | 68.2 | 96.4 | 94.1 | 38.1 | - | - | 18.8 | 11.1 | <0.01 |
| Confectionery | 66.9 | 100 | 76.5 | 9.5 | - | - | 100 | 33.3 | <0.01 |
| Alcoholic drinks | 76.8 | 100 | 98.5 | 33.3 | 16.7 | - | 56.3 | 44.4 | <0.01 |
| *ANOVA test. | | | | | | | | | |

**Table A3. Comparison between pairs of ‘healthier-less healthy’ food items.**

| **Food item** | **Availability (% of retailers)** | | | | **Price (€/kg or €/L), median [IQR]** | | | |
| --- | --- | --- | --- | --- | --- | --- | --- | --- |
|  | **Healthier** | **Less healthy** | **Difference** | **p-value*** | **Healthier** | **Less healthy** | **Difference** | **p-value**** |
| Nuts (unprocessed vs. salty) | 55.6 | 69.5 | -13.9 | 0.01 | - | - | - | - |
| Juice (100% vs. not 100%) | 37.1 | 73.5 | -36.4 | <0.01 | 1.30 [1.10] | 1.32 [1.15] | -0.02 | 0.01 |
| Cola drink (light vs. regular) | 75.5 | 74.8 | 0.7 | 0.89 | 2.10 [0.69] | 2.10 [0.69] | 0.00 | 0.16 |
| Cereals (low sugar vs. regular) | 31.8 | 43.0 | -11.2 | 0.04 | 4.13 [3.13] | 5.45 [4.29] | -1.32 | 0.73 |
| Milk (skimmed vs. whole) | 71.5 | 72.2 | -0.7 | 0.90 | 1.00 [0.25] | 1.00 [0.25] | 0.00 | 0.04 |
| Cheese (cream vs. semi-hard) | 43.7 | 58.3 | -14.6 | 0.01 | - | - | - | - |
| Oil (olive vs. sunflower) | 53.6 | 65.6 | -12.0 | 0.03 | 4.70 [1.88] | 1.59 [0.73] | 3.11 | <0.01 |
| Butter (salt-free vs. regular) | 35.8 | 50.3 | -14.5 | 0.01 | - | - | - | - |
| Rice (whole vs. white) | 17.2 | 68.2 | -51.0 | <0.01 | 1.65 [0.76] | 1.30 [0.79] | 0.35 | <0.01 |
| Meat (chicken vs. beef) | 19.9 | 19.9 | 0.0 | 1.00 | 6.90 [1.11] | 9.99 [3.12] | -3.09 | <0.01 |
| Frozen fish (unprocessed vs. processed) | 20.5 | 18.5 | 2.0 | 0.66 | - | - | - | - |
| *Two sample test of proportions. **Wilcoxon matched-pairs signed-rank test. | | | | | | | | |

**Table A4. NEMS-S-MED score by type of retailer.**

| **Type of retailer** | **NEMS-S-MED Score (median [IQR])** | | |
| --- | --- | --- | --- |
|  | **Total (0-49)** | **Availability (0-37)** | **Price (0-12)** |
| Supermarket (n=28) | 37.5 [12.5] | 32 [12] | 5 [2] |
| Convenience store (n=68) | 13.5 [6] | 11 [5.5] | 3 [1] |
| Fruit and vegetable store (n=21) | 12 [4] | 11 [3] | 0 [1] |
| Butcher's' (n=6) | 4.5 [3] | 3.5 [1] | 1 [2] |
| Fishmonger's' (n=3) | 6 [3] | 6 [3] | 0 [0] |
| Bakery (n=16) | 2 [5] | 1 [4.5] | 0 [2] |
| Other (n=9) | 9 [20] | 5 [18] | 0 [2] |
| **Total (n=151)** | **13 [12]** | **11 [9]** | **3 [3]** |

**Table A5. Selling price of individual items and Relative Price Index by store type.**

| **Food item** | **Type of food retailer** | | | | | | | **p-value*** |
| --- | --- | --- | --- | --- | --- | --- | --- | --- |
|  | **Supermarket (n=28)** | **Convenience (n=68)** | **Fruit store (n=21)** | **Butchers (n=6)** | **Fishmongers (n=3)** | **Bakery (n=16)** | **Other**  **(n=9)** |  |
| Apples | 1.59 [0.50] | 2.05 [1.20] | 1.74 [1.00] | - | - | - | 3.19 [3.01] | 0.01 |
| Tomatoes | 1.24 [0.46] | 1.89 [0.81] | 1.29 [0.89] | - | - | - | 1.59 [2.15] | 0.07 |
| Frozen spinach | 1.80 [1.77] | 2.94 [1.13] | 4.50 [0.00] | - | - | - | 6.38 [0.00] | 0.03 |
| Juice 100% | 1.08 [0.73] | 1.80 [1.05] | 1.20 [1.75] | - | - | - | 2.75 [1.56] | 0.98 |
| Juice not 100% | 0.74 [0.80] | 1.50 [0.75] | 2.56 [6.02] | - | - | 1.30 [6.20] | 5.49 [3.03] | 0.11 |
| Light cola drink | 1.71 [1.08] | 2.25 [0.30] | 2.33 [0.30] | - | - | 2.40 [0.30] | 3.15 [1.50] | <0.01 |
| Regular cola drink | 1.71 [1.08] | 2.25 [0.30] | 2.25 [0.15] | - | - | 2.40 [0.30] | 2.18 [0.45] | <0.01 |
| Whole bread | 3.30 [2.21] | 3.51 [1.72] | 4.75 [1.50] | - | - | 7.53 [3.94] | 7.60 [0.00] | 0.72 |
| Low sugar cereals | 3.94 [2.58] | 5.90 [3.84] | 9.95 [0.00] | - | - | - | 5.35 [4.20] | 0.17 |
| Regular cereals | 3.66 [3.20] | 6.91 [4.17] | 7.87 [0.00] | - | - | - | 3.73 [0.00] | <0.01 |
| Skimmed milk | 0.61 [0.26] | 1.00 [0.10] | 1.10 [0.70] | - | - | 1.00 [0.08] | 1.50 [0.00] | <0.01 |
| Whole milk | 0.61 [0.25] | 1.00 [0.10] | 1.10 [0.70] | - | - | 1.00 [0.08] | 1.50 [0.00] | <0.01 |
| Olive oil | 3.90 [0.91] | 4.95 [1.45] | 4.60 [1.03] | - | - | 4.50 [26.55] | 8.20 [0.00] | 0.03 |
| Sunflower oil | 1.21 [0.43] | 1.80 [0.50] | 1.55 [0.30] | - | - | 1.80 [1.00] | 10.00 [15.80] | <0.01 |
| Whole rice | 1.60 [0.30] | 1.95 [0.30] | 1.50 [0.00] | - | - | - | 6.20 [0.00] | <0.01 |
| White rice | 0.79 [0.67] | 1.45 [0.70] | 1.25 [0.88] | - | - | 1.43 [0.47] | 7.00 [0.00] | <0.01 |
| Chicken | 6.93 [1.46] | 9.22 [0.00] | - | 6.24 [1.18] | - | - | 7.10 [0.00] | 0.16 |
| Beef | 9.98 [2.59] | 1.59 [0.00] | - | 11.99 [2.60] | - | - | 7.80 [0.00] | 0.02 |
| Hake | 9.38 [3.88] | - | - | - | 8.90 [9.90] | - | - | 0.89 |
| **Relative Price Index** | **0.83 [0.33]** | **1.01 [0.27]** | **0.98 [0.35]** | **1.04 [0.37]** | **0.78 [0.86]** | **0.97 [1.66]** | **1.30 [0.54]** | **<0.01** |

| Prices expressed in €/kg or €/L. Numbers are median [Interquartile range]. *ANOVA. |
| --- |

**Table A6. Price comparison between supermarkets and convenience stores.**

| **Price of food item (€ per kg/L)** | **Type of food retailer** | | **p-value*** |
| --- | --- | --- | --- |
|  | **Supermarket (n=28)** | **Convenience (n=66)** |  |
| Apples | 1.59 [0.50] | 2.05 [1.20] | 0.05 |
| Tomatoes | 1.24 [0.46] | 1.89 [0.81] | 0.05 |
| Frozen spinach | 1.80 [1.77] | 2.94 [1.13] | 0.57 |
| Juice 100% | 1.08 [0.73] | 1.80 [1.05] | 0.91 |
| Juice not 100% | 0.74 [0.80] | 1.50 [0.82] | 0.47 |
| Light cola drink | 1.71 [1.08] | 2.25 [0.30] | <0.01 |
| Regular cola drink | 1.71 [1.08] | 2.25 [0.30] | <0.01 |
| Whole bread | 3.30 [2.21] | 3.51 [1.72] | 0.59 |
| Low sugar cereals | 3.94 [2.58] | 5.90 [3.84] | 0.18 |
| Regular cereals | 3.66 [3.20] | 6.91 [4.17] | <0.01 |
| Skimmed milk | 0.61 [0.26] | 1.00 [0.10] | <0.01 |
| Whole milk | 0.61 [0.25] | 1.00 [0.10] | <0.01 |
| Olive oil | 3.90 [0.91] | 5.00 [1.45] | 0.69 |
| Sunflower oil | 1.21 [0.43] | 1.80 [0.50] | 0.05 |
| Whole rice | 1.60 [0.30] | 1.95 [0.30] | 0.29 |
| White rice | 0.79 [0.67] | 1.45 [0.70] | <0.01 |
| Chicken | 6.93 [1.46] | 9.22 [0.00] | 0.07 |
| Beef | 9.98 [2.59] | 1.59 [0.00] | 0.01 |
| Hake | 9.38 [3.88] | - | - |
| **Price index** | **0.83 [0.33]** | **1.02 [0.26]** | **<0.01** |
| Median [Interquartile range]. *ANOVA test. | | | |

**Table A7. Price comparison between supermarkets and fruit stores.**

| **Price of food item (€ per kg/L)** | **Type of food retailer** | | **p-value*** |
| --- | --- | --- | --- |
|  | **Supermarket (n=28)** | **F&Vstore (n=21)** |  |
| Apples | 1.59 [0.50] | 1.74 [1.00] | 0.54 |
| Tomatoes | 1.24 [0.46] | 1.29 [0.89] | 0.54 |
| Frozen spinach | 1.80 [1.77] | 4.50 [0.00] | 0.12 |
| Juice 100% | 1.08 [0.73] | 1.20 [1.75] | 0.74 |
| Juice not 100% | 0.74 [0.80] | 2.56 [6.02] | 0.09 |
| Light cola drink | 1.71 [1.08] | 2.33 [0.30] | 0.02 |
| Regular cola drink | 1.71 [1.08] | 2.25 [0.15] | 0.06 |
| Whole bread | 3.30 [2.21] | 4.75 [1.50] | 0.92 |
| Low sugar cereals | 3.94 [2.58] | 9.95 [0.00] | 0.02 |
| Regular cereals | 3.66 [3.20] | 7.87 [0.00] | 0.14 |
| Skimmed milk | 0.61 [0.26] | 1.10 [0.70] | 0.01 |
| Whole milk | 0.61 [0.25] | 1.10 [0.70] | 0.01 |
| Olive oil | 3.90 [0.91] | 4.60 [1.03] | 0.79 |
| Sunflower oil | 1.21 [0.43] | 1.55 [0.30] | 0.21 |
| Whole rice | 1.60 [0.30] | 1.50 [0.00] | 0.67 |
| White rice | 0.79 [0.67] | 1.25 [0.88] | 0.06 |
| Chicken | 6.93 [1.46] | - | - |
| Beef | 9.98 [2.59] | - | - |
| Hake | 9.38 [3.88] | - | - |
| **Price index** | **0.83 [0.33]** | **0.98 [0.35]** | **0.30** |
| Median [Interquartile range]. *ANOVA test. | | | |

**Figure S1. Area-level socioeconomic status in the city of Madrid.**
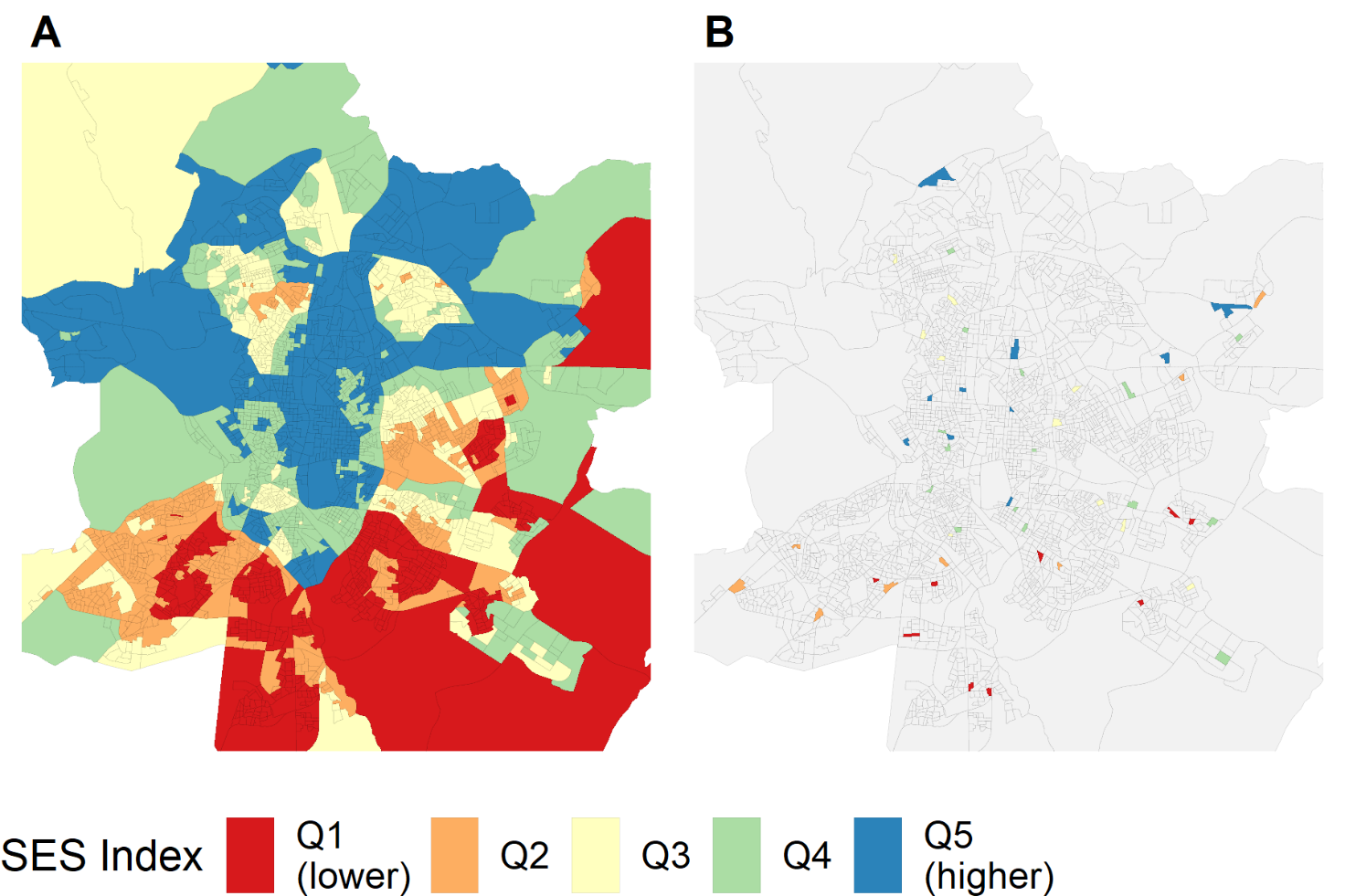


**A: census tracts of the city of Madrid (n=2443). B: census tracts assessed in our sample (n=52).** SES index is constructed from seven indicators of education, employment, and housing prices. Quintiles of SES range from Q1 = most disadvantaged, to Q5 = most advantaged. Data from 2017.

**Figure S2. Flow diagram of food stores included in the study (*n* = 151)**
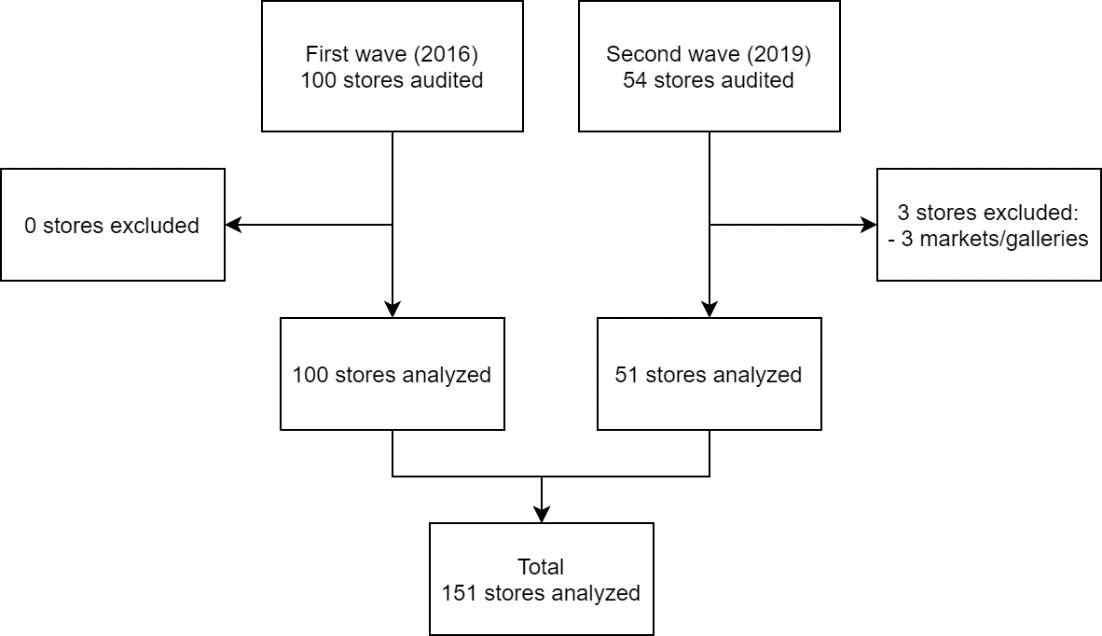

Supplement: Supplementary file 1 [file S1368980022002348sup001.docx]
